# Editorial Note: Repeatability and reproducibility of eight macular intra-retinal layer thicknesses determined by an automated segmentation algorithm using two SD-OCT instruments

**DOI:** 10.1371/journal.pone.0324282

**Published:** 2025-05-12

**Authors:** The PLOS One Editors

The *PLOS One* Editors issue this Editorial Note to inform readers about a concern with this article’s [1] references. Specifically, it appears that the “Measurements of Macular Thickness of Intra-Retinal Layers” section of the Materials and Methods is missing a reference to Chiu et al. [2] which should have been included in the article.
